# Microbial community structure is stratified at the millimeter-scale across the soil–water interface

**DOI:** 10.1038/s43705-022-00138-z

**Published:** 2022-06-30

**Authors:** Yu-Jia Cai, Zi-Ao Liu, Sha Zhang, Hao Liu, Graeme W. Nicol, Zheng Chen

**Affiliations:** 1 Department of Health and Environmental Sciences, Xi’an Jiaotong-Liverpool University, 111 Ren’ai Road, Suzhou, Jiangsu 215123 China; 2 Department of Geography & Planning, School of Environmental Sciences, University of Liverpool, Brownlow Hill, Liverpool, L697ZX UK; 3 Univ Lyon, CNRS, INSA Lyon, Université Claude Bernard Lyon 1, Ecole Centrale de Lyon, Ampère, UMR5005, 69134 Ecully cedex, France

**Keywords:** Microbial ecology, Biogeochemistry, Biogeochemistry

## Abstract

Soil–water interfaces (SWI) are biogeochemical hotspots characterized by millimeter-scale redox gradients, indicating that parallel changes are also present in microbial community structure and activity. However, soil-based analyses of microbial community structure typically examine bulk samples and seldom consider variation at a scale relevant to changes in environmental conditions. Here we presented a study that aimed to describe millimeter-scale variance in both microbial community structure and physicochemical properties in a lab flooded soil. At this fine-scale resolution, the stratification of biogeochemical properties (e.g., redox potential, nitrate concentration) was consistent with the structure of the active microbial community with clear shifts in the relative abundance of transcriptionally active populations associated with changing redox conditions. Our results demonstrate that spatial scale should be carefully considered when investigating ecological mechanisms that influence soil microbial community structures.

The soil–water interface (SWI) is a biogeochemical hotspot where aerobic microbes are enriched at the top and respire oxygen from the surface water, with microbes capable of anaerobic respiration residing toward the bottom [1]. Physicochemical gradients across the SWI create a range of niches for the dwelling microbes [2] and therefore make the SWI an ideal model system to investigate links between microbial community structure and ecosystem processes. However, the microbial community structure across the SWI remains poorly understood.

Environmental filtering, i.e., the shaping of soil microbial community structure by abiotic factors, is considered an important factor influencing microbial community assembly in highly heterogeneous environments [3, 4]. Although the physicochemical gradients across the SWI usually occur at the μm or mm scale, a stratified microbial community is rarely observed, largely limited by the sampling approaches and analytical methods used. In many cases, the interface is sliced into ~1 cm layer to obtain sufficient material for microbiome analysis [5, 6], but these layers are typically too thick to allow determination of physicochemical gradients with any precision. We, therefore, propose that typical centimeter-scale or bulk sampling hinders attempts to adequately characterize patterns of microbial community structure across the SWI. Furthermore, considering that not all microbes are active in any given environment, microbial community structure profiled by the analysis of genomic DNA does not necessarily reflect the active microbes or community assembly processes [7, 8]. We hypothesized that stratification of active microbial community structures would be observed across the SWI if sampled and analyzed appropriately i.e., at the mm-scale using an RNA-based approach.

We conducted a soil incubation experiment for mm-scale sampling (Fig. S1) using analysis of rRNA content to profile the metabolically active members of the microbial community. Specifically, soil was flooded with sterilized deionized water and incubated for 20 days at 25 °C in the dark before a 50 mm soil core was taken and sampled at 2 mm intervals with prokaryote communities profiled using amplicons derived from reverse-transcribed 16S rRNA (detailed in Supplementary Information). After processing and taxonomic assignment of sequences, distance decay relationships (DDR) describing the similarity in community structures over distance were performed [9, 10]. We detected a strong DDR with the bacterial community similarity (1- Canberra dissimilarity) [9] significantly decreasing with distance through the SWI (Mantel *r* = 0.547, *p* < 0.001) (Fig. 1A). While DDR has been extensively used in microbiome research, patterns vary depending on the sampling scale. Microbial DDR patterns are assumed weak due to the high abundance, short life cycle, and dispersal abilities of microbes [9, 11]. In this study, we observed a DDR that was stronger than that of larger sampling scales (e.g., m or km) [9], but comparable to the cm scale [10]. This demonstrates that the spatial organization of soil microbes with relatively high community dissimilarity can be observed with appropriate spatial scales of analysis [9, 12, 13].

**Fig. 1:**
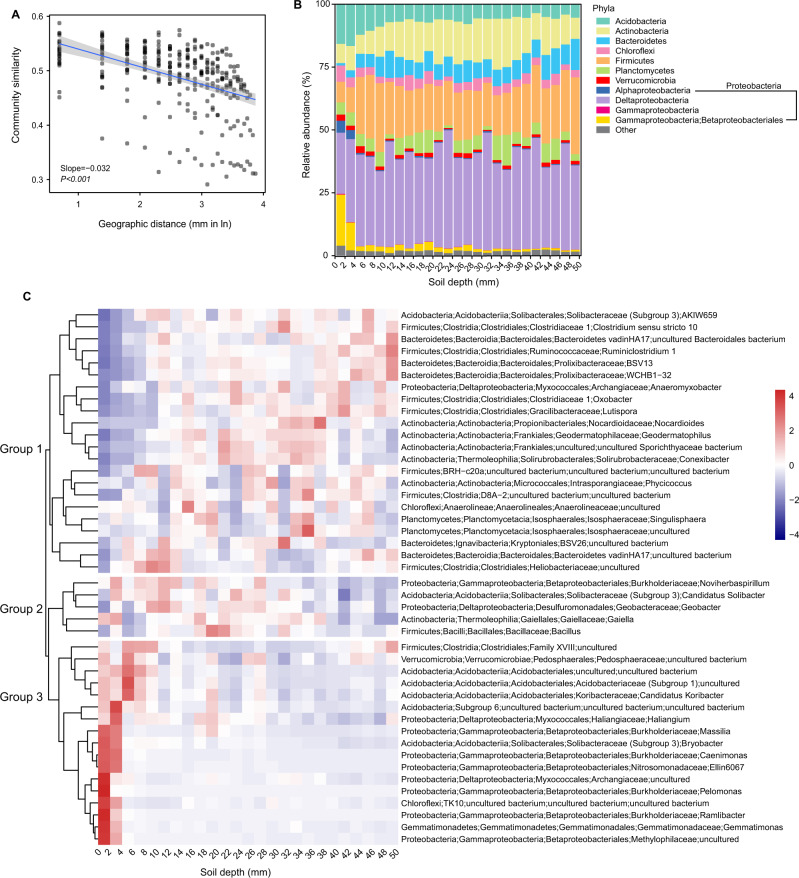
Community composition of the active microbial communities based on relative abundance of 16S rRNA gene transcripts through a 50 mm water–soil interface. **A** Changes in Bray-Curtis similarities of microbial communities with spatial distance. A linear trend was determined using ordinary least-squares linear regression with the shaded area representing 95% confidence intervals. **B** Relative abundance of phyla. Only those with a relative abundance ≥1% were included, while those <1% categorized as “Other”. **C** Heatmap showing the relative abundance difference of the selected 44 bacterial genera (relative abundance ≥1%). An increase in abundance tends toward red, while a decrease tends toward blue.

Distinct patterns of active bacterial communities were observed at the phylum level across the SWI (Fig. 1B). For example, the relative abundance of rRNA reads from the *Betaproteobacteriales* within the *Gammaproteobacteria* class (a modification in the SILVA 132 database) decreased greatly (27-fold difference at the extremes of the SWI), while *Bacteroidetes* increased with soil depth (13-fold difference at the extremes). We then examined the active bacterial community composition at the family and genus level for a higher taxonomic resolution (Figs. S2 and 1C). Sequences representing 44 bacterial genera (each with a relative abundance ≥1%) accounted for 77% of all total sequence and were clustered into three different groups reflecting different distribution patterns (Fig. 1C). Bacterial genera within group 1 generally had their lowest relative abundance of 16S rRNA transcripts in the top 0–6 mm and were dominated by strict or facultative anaerobic bacteria such as representatives of the *Ruminococcaceae* family, *Anaeromyxobacter* and *Oxobacter* genera. Bacteria in group 2 were more active from 2 to 40 mm, generally showed low relative activity only at the deepest layers of 40–50 mm and consisted of aerobic and facultative anaerobic bacteria including *Bacillus* and *Gaiella* representatives. In contrast to groups 1 and 2, bacteria in group 3 exhibited a clear decrease in their relative abundance from the surface to 50 mm in the flooded soil profile. Most of the bacteria in this group had the highest relative abundance of 16S rRNA transcripts in the first 4 mm topsoil layer, indicating a cluster of strictly aerobic organisms including representatives of the genera *Massilia* and *Gemmatimonas*.

We measured a range of soil physicochemical properties (Fig. 2A–E) and performed tbRDA analysis based on the selected 44 bacterial genera (Fig. 2F). The measured physicochemical properties correlated with 80.0% of the variance of the selected taxa (Fig. 2F). The main correlating factors were redox potential (*F* = 29.6, *P* < 0.001) and nitrate concentration (*F* = 50.3, *P* < 0.001). Consistent with the distribution of bacteria across the SWI (Figs. 1C and 2), variance in redox potential could be divided into oxic, transition, and anoxic zones (Fig. 2A). The vertical stratification of the microbial community was primarily driven by the availability of oxygen rather than that of other common electron acceptors, as generally observed in lake and marine sediments [6, 14–16].

**Fig. 2:**
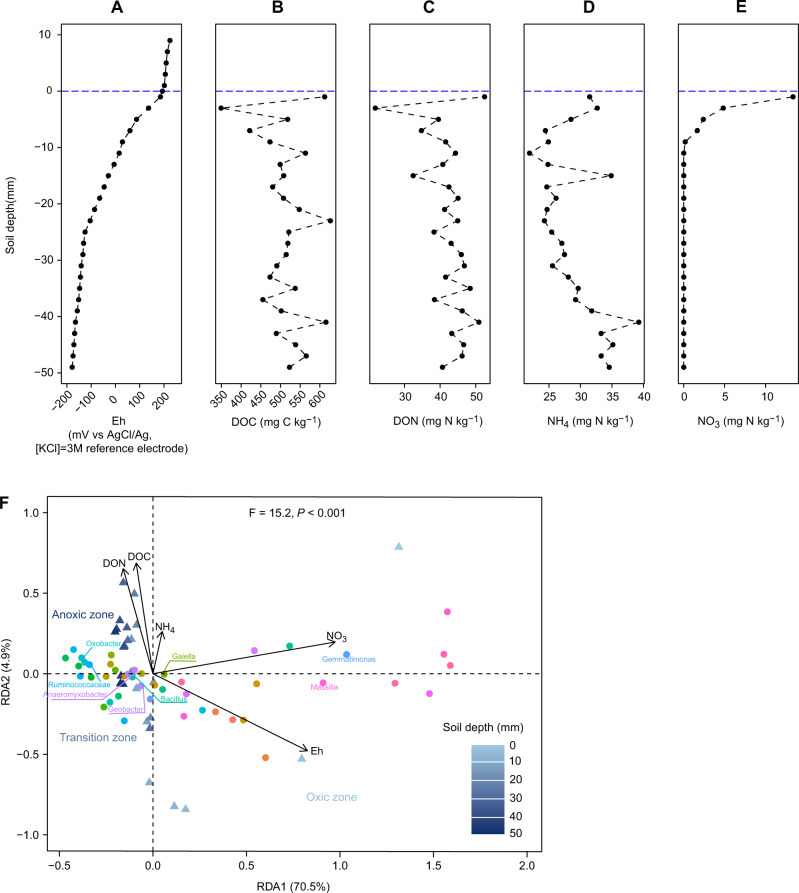
Depth profiles of soil physicochemical properties and microbial community structure through the SWI depth profile. **A** Redox potential. **B** Dissolved organic carbon (DOC). **C** Dissolved organic nitrogen (DON). **D** Ammonium-N (NH_4_-N). **E** Nitrate-N (NO_3_-N). **F** Distance based-redundancy analysis (dbRDA) triplot based on the selected 44 bacterial genera (relative abundance ≥1%) across the SWI. Triangles, bacterial genera; circles, soil samples across SWI; arrows, environmental factors.

Microbial communities were functionally annotated using FAPROTAX [17]. While the prediction of metabolic attributes from 16 S rRNA sequences must be interpreted with caution, the results of functional group distribution were nevertheless consistent with the gradient of biogeochemical processes across the SWI. For example, transcripts associated with organisms performing oxygen-dependent nitrification processes of ammonia and nitrite oxidation were most abundant within the first 4 mm and decreased as soil depth increased (Fig. S3a). This decrease was consistent with the nitrate profile in which nitrate was not detectable below 8 mm (Fig. 2E). Similarly, the abundance of transcripts associated with organisms performing aerobic methanol oxidation and methylotrophy decreased rapidly as soil depth increased (Fig. S3). The methanotrophic populations demonstrated high activity at oxic–anoxic interface [18, 19], and was consistent with the changes in the transcript number of particulate methane monooxygenase sub-unit A (*pmoA*) gene transcripts, a functional marker for methane-oxidizing bacteria) (Fig. S3b). Other metabolic activities such as iron respiration and fermentation were mainly distributed in the sub-oxic areas as these metabolic processes are performed in the absence of oxygen.

In summary, a stratified active microbial community could be observed through the SWI at the mm-scale. These findings could direct future biogeochemical research and identification of corresponding microbial populations across the SWI as these processes occur at very narrow zones and further affect large-scale ecosystem responses (e.g., methane fluxes) [1, 20]. “Who is doing what” remains a major challenge in microbial ecology. We believe the isolation of the specific strains corresponding to a targeted process would be more successful if the inoculum was sampled from hot spots of activity identified via a high resolution, mm-scale profiling of the microbial community. The method could further be applied to study microbial responses to environmental stress (e.g., pollutants, toxics) and allows characterization and enrichment of uncultivated strains with previously unknown metabolic functions by creating artificial gradients. We conclude that the analysis of microbial communities at an appropriate spatial scale should facilitate the identification of the contributors to specific functions processed in microbiome research.

## Supplementary information

Supplementary Material
