# Supplementary material for: Microbial community structure is stratified at the millimeter-scale across the soil–water interface
Source: ISME Commun. 2022 Jun 30;2:53. doi: 10.1038/s43705-022-00138-z (PMC9723559; doi:10.1038/s43705-022-00138-z)
Supplement: Supplementary file 1 — Supplementary Material [file 43705_2022_138_MOESM1_ESM.docx]

**Supplementary Material**

Microbial community structure is stratified at the millimeter-scale across the soil-water interface

Yu-Jia Cai^1,2^, Zi-Ao Liu^1^, Sha Zhang^1,2^, Hao Liu^1,2^, Graeme W Nicol^3^, Zheng Chen^1^*

^1^ Department of Health and Environmental Sciences, Xi’an Jiaotong-Liverpool University, 111 Ren’ai Road, Suzhou, Jiangsu, 215123, China

^2^ Department of Geography & Planning, School of Environmental Sciences, University of Liverpool, Brownlow Hill, Liverpool, L697ZX, United Kingdom

^3^ Univ Lyon, CNRS, INSA Lyon, Université Claude Bernard Lyon 1, Ecole Centrale de Lyon, Ampère, UMR5005, 69134 Ecully cedex, France

*Corresponding author: Zheng Chen; Tel: +86-(0)512-81880471; Fax: +86-(0)512-88161899; Email: ebiogeochem@outlook.com, [Zheng.Chen@xjtlu.edu.cn](mailto:Zheng.Chen@xjtlu.edu.cn)

**Materials and methods**

**Soil incubation experiment**

The soil incubation experiment was conducted to investigate the active microbial community during the flooding of soils. The paddy soils used in this study were collected from Shaoguan (25°6'N, 113°38'E), South China in 2017. Briefly, 0-20 cm layer of soils were sampled and transported to the lab within 48 h. The soils were air-dried and sieved through 2 mm with plant debris and gravel removed. Soil pH was measured in a suspension of soil and CO_2_-free deionized (DI) water (1: 2.5, w: v) with a pH analyzer. Soil dissolved organic carbon and nitrogen (DOC, DON) were extracted with 0.5 M K_2_SO_4_ (1:5, w: v) and determined using a C/N analyzer (Jena, Germany). Soil total Fe and Mn were extracted by NHO_3_ (electronic grade) and H_2_O_2_ (A.R.) and determined using Inductively Coupled Plasma Mass Spectrometry (ICP-MS) (Perkin Elmer, USA). The tested soils was acidic (pH = 5.55) and contained 136.94 mg/kg DOC, 23.20 mg/kg DON, 60.84 g/kg Fe and 290.6 mg/kg Mn dry soil, respectively.

Soil was added into a lightproof plastic pot (mean inner diameter × height =15×17 cm) to form a 15-cm soil layer (Fig. S1). Sterilized DI water was then added to flood the soil with a 5-cm layer of standing water remaining above the soil surface. The soil was incubated at 25 °C for 20 days with a black lid to avoid light. The incubated soils were checked every 2 day to maintain the standing water layer. On day 20, two soil cores were sampled and freshly sliced for chemical and microbial community analysis, respectively. Before sampling, the redox potential (Eh) profile was determined using an Eh microelectrode.

**Soil core sampling and sectioning**

The soil core was sampled using a sterile 50-mL syringe column and was carefully inserted into the soil so that the soil-water interface (SWI) was not disturbed (Fig. S1a). The syringe column was slightly rotated before removing the core (Fig. S1b). A hole was then drilled to remove the surface water. Thus, a 50-mm soil core was sampled with minimized disturbance of the soil (Fig. S1c).

To determine the spatial distribution of active microbial populations throughout the SWI, thin sections of the soil samples were prepared for soil total RNA extraction and chemical extraction. After soil core sampling, the soil core was vertically pushed out at 2 mm intervals and sliced with a sterile ceramic knife. The sliced soil sections were immediately processed for soil RNA extraction and chemical extraction (Fig. S1d).

**Soil solid phase analysis**

The 2-mm sectioned soil samples were extracted with 5 mL 0.5M K_2_SO_4_. The extract were filtered through 0.45 μm and diluted for DOC and DON analysis. Ammonium and nitrate concentrations were determined in microscale samples using established colorimetric assays [1]. Spectrophotometric determinations were made at 660 and 560 nm for ammonium and nitrate, respectively.

**Soil RNA extraction, PCR and high throughput sequencing**

Soil total RNA was extracted using the PowerSoil Total RNA Isolation Kit (MO BIO, USA) according to the manufacturer’s instructions. RNA quality and concentration were assessed by a NanoDrop ND-2000 spectrophotometer (Thermo Scientific, USA). Removal of genomic DNA and reverse transcription were performed with the PrimeScript RT reagent kit with gDNA Eraser (TAKARA, China).

The V4 region of bacterial 16S rRNA was amplified by PCR using the primer set 515F/806R [2]. PCR mixture and sequencing were as described previously [3]. Briefly, the PCR mixture contained 4 μL of 5 × FastPfu Buffer, 2 μL of 2.5mM dNTPs, 0.8 μL of each primer (5 μM), 0.4 μL of FastPfu Polymerase, and 10ng of total cDNA in ABI GeneAmp 9700. Each sample was performed with three technical replicates. The PCR products were purified and pooled. Paired-end sequencing (2 × 250 bp) was performed using the Illumina MiSeq platform (Majorbio Bio-Pharm Technology Co. Ltd., Shanghai, China).

**Quantification of *pmoA* gene transcript abundance**

The primer set A189F (5’-GGNGACTGGGACTTCTGG-3’)/ mb661r (5’- CCGGMGCAACGTCYTTACC-3’) [4] was used to quantify the transcript abundance of *pmoA*, a functional marker for methane oxidizing bacteria. Transcript number of *pmoA* gene was quantified using quantitative PCR with three technical replicates per sample using a Light Cycler (Light Cycler 480 II, Roche, Switzerland). The mixture for the qPCR assay consisted of 10 μL TB Green premix (TAKARA), 0.5 μL for forward and reverse primer (10 μM), 1.0 μL total cDNA and 8 μL DNase-free water. The qPCR thermal program was as follows: 2 min at 94 °C, followed by 40 cycles of denaturation at 94 °C for 45 s, 53 °C for 45 s, 72 °C for 55s. The fluorescence intensity was read at 83 °C. For the quantification of the target gene, a standard curve was generated from serial dilutions of plasmid DNA containing a fragment of the *pmoA* gene. The amplification efficiency of the standard plasmid DNA was 98% ~103% and the logarithmic correlation (R^2^) of the standard curve was higher than 0.99. Melting curve and gel electrophoresis were conducted to check the specificity of qPCR products.

**Bioinformatics analysis**

Raw sequences were processed in QIIME 1.9.1 (Quantitative Insight into Microbial Ecology) pipeline [5, 6]. To detect and filter chimeras, UPARSE was applied to bin the sequences into OTUs at the 97% sequence identity level [7]. Representative sequences were compared against the SILVA (Release 132) reference alignment using the RDP classifier [8]. In SILVA release 132, *Betaproteobacteria* is now considered as an order of *Gammaproteobacteria*. After quality filtering and chimeric removal, a total of 1 932 744 sequences (50 788 ~ 92 704 sequences per sample) were obtained from the 25 sectioned soil samples. As an even depth of sampling is required for further analysis, all the samples were randomly rarefied to 50 000 sequences generating 5 039 OTUs. The bacterial OTUs were annotated and assigned to 19 phyla and 303 genera. The relative abundance of 11 phyla/classes were higher than 1% in most samples accounting for 98% of the total sequences. Sequence reads were deposited into the NCBI Sequence Read Archive (SRA) database with the project accession number PRJNA826420. FAPROTAX 1.2.4 [9] was used to predict the metabolic functions of the microbial community. FAPROTAX [9] database maps the taxonomic microbial community profiles into putative metabolic or other ecologically relevant functions based on cultured microbes. The bacterial functional groups were categorized into metal related, C, N and S cycling.

**Statistical analyses**

The R platform [10] was adopted to perform the statistical analyses. A distance decay relationship was described as the decreasing connection in community similarity with increasing spatial distance [11] and estimated by regressing community similarity against pairwise geographic distance [12, 13]. Canberra dissimilarity was chosen for its incorporation of abundance data and sensitivity to rare species within communities [13]. Community dissimilarity was calculated using the “vegdist” function in the ‘vegan’ package [14]. A Mantel test was then conducted to test the significance between community similarity and geographic distance in R. To reveal the relationships between microbial community structure and environmental factors, we used the selected bacterial genera in Fig. 1c to perform a distance based-redundancy analysis using the “rda” function in the ‘vegan’ package. Monte Carlo permutation test was used to test the statistical significance of the constrained ordination (Fig. 2f). For the selected taxa ordination, triplot was generated using the ‘ggplot2’ package and Adobe Illustrator.

**References**

1. Sims GK, Ellsworth TR, Mulvaney RL. Microscale determination of inorganic nitrogen in water and soil extracts. Commun Soil Sci Plant Anal. 2008; 26:303–16.

2. Hugerth LW, Wefer HA, Lundin S, Jakobsson HE, Lindberg M, Rodin S, et al. DegePrime, a program for degenerate primer design for broad-taxonomic-range PCR in microbial ecology studies. Appl Environ Microbiol. 2014; 80:5116-23.

3. Cai Y, Shen JP, Di HJ, Zhang LM, He JZ. Variation of soil nitrate and bacterial diversity along soil profiles in manure disposal maize field and adjacent woodland. J Soils Sediments. 2020; 20:3557-68.

4. Costello  AM, Lidstrom ME. Molecular characterization of functional and phylogenetic genes from natural populations of methanotrophs in lake sediments. Appl Environ Microbiol. 1999; 65:5066-74.

5. Caporaso JG, Lauber CL, Walters WA, Berg-Lyons D, Lozupone CA, Turnbaugh PJ, et al. Global patterns of 16S rRNA diversity at a depth of millions of sequences per sample. Proc Natl Acad Sci USA. 2011; 108:4516-22.

6. Caporaso JG, Kuczynski J, Stombaugh J, Bittinger K, Bushman FD, Costello EK, et al. QIIME allows analysis of high-throughput community sequencing data. Nat Methods. 2010; 7:335-6.

7. Edgar RC. UPARSE: highly accurate OTU sequences from microbial amplicon reads. Nat Methods. 2013; 10:996-8.

8. Quast C, Pruesse E, Yilmaz P, Gerken J, Glckner FO. The SILVA ribosomal RNA gene database project: improved data processing and web-based tools. Nucleic Acids Res. 2013; 41:D590-6.

9. Louca S, Parfrey LW, Doebeli M. Decoupling function and taxonomy in the global ocean microbiome. Science. 2016; 353:1272-7.

10. Team R Core. R: A language and environment for statistical computing. R Foundation for Statistical Computing, Vienna, Austria, 2018.

11. Xue R, Zhao KK, Yu XL, Stirling E, Liu S, Ye SD, Ma B, et al. Deciphering sample size effect on microbial biogeographic patterns and community assembly processes at centimeter scale. Soil Biol Biochem. 2021; 156:108218.

12. Nekola JC, White PS. The distance decay of similarity in biogeography and ecology. J Biogeogr. 1999; 26:867-78.

13. Meyer KM, Memiaghe H, Korte L, Kenfack D, Alonso A, Bohannan BJM. Why do microbes exhibit weak biogeographic patterns? ISME J. 2018; 12:1404-13.

14. Dixon P. VEGAN, a package of R functions for community ecology. J Veg Sci. 2003; 14:927-30.


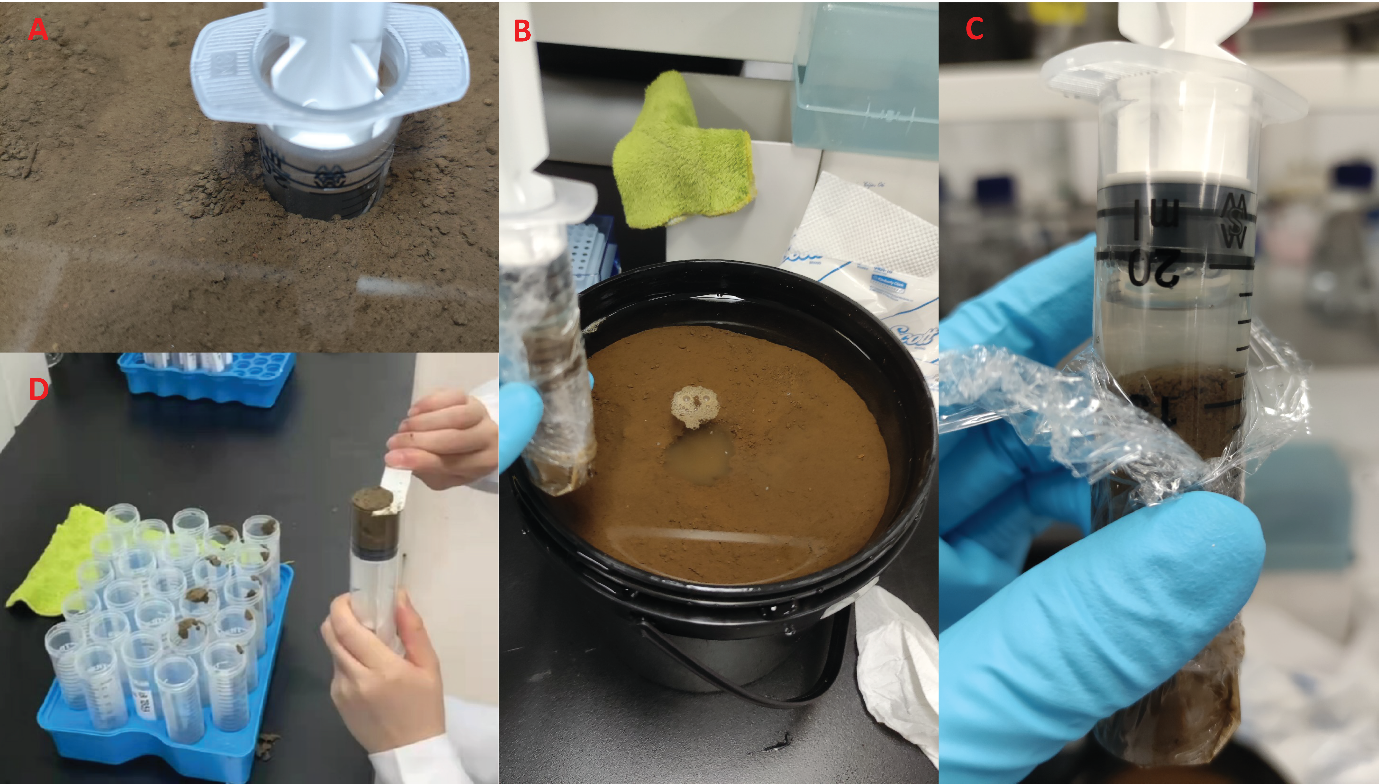


**Figure S1.** Soil core sampling and slicing. A) and B) Soil core sampling process. C) A 50-mm soil core sample with the soil-water interface well preserved. D) Soil sectioning for chemical analysis.


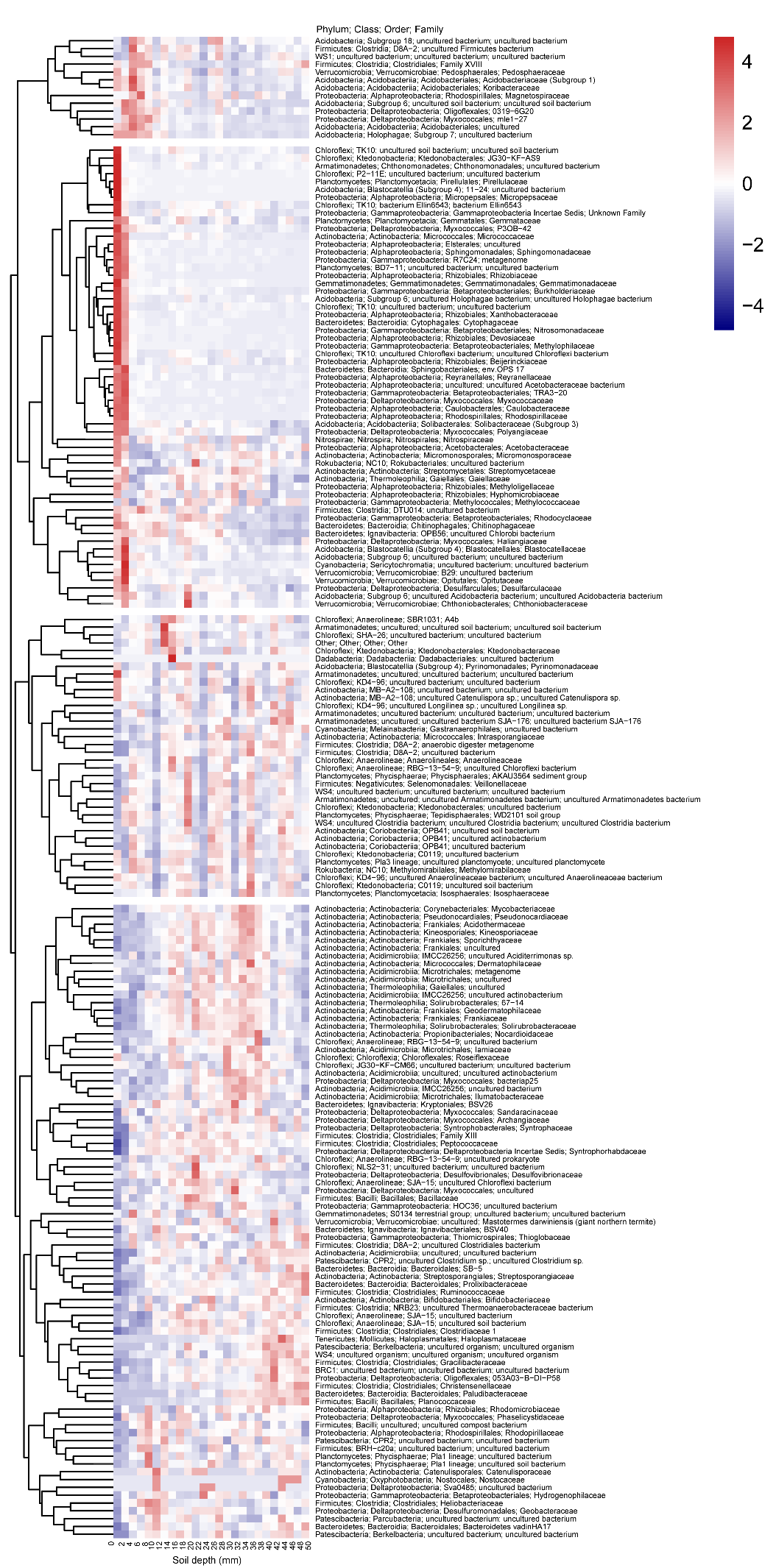


**Figure S2.** Heatmap showing the relative abundance difference of the bacterial community at the family level across the soil-water interface. An increase in abundance tends toward red, while a decrease tends toward blue.


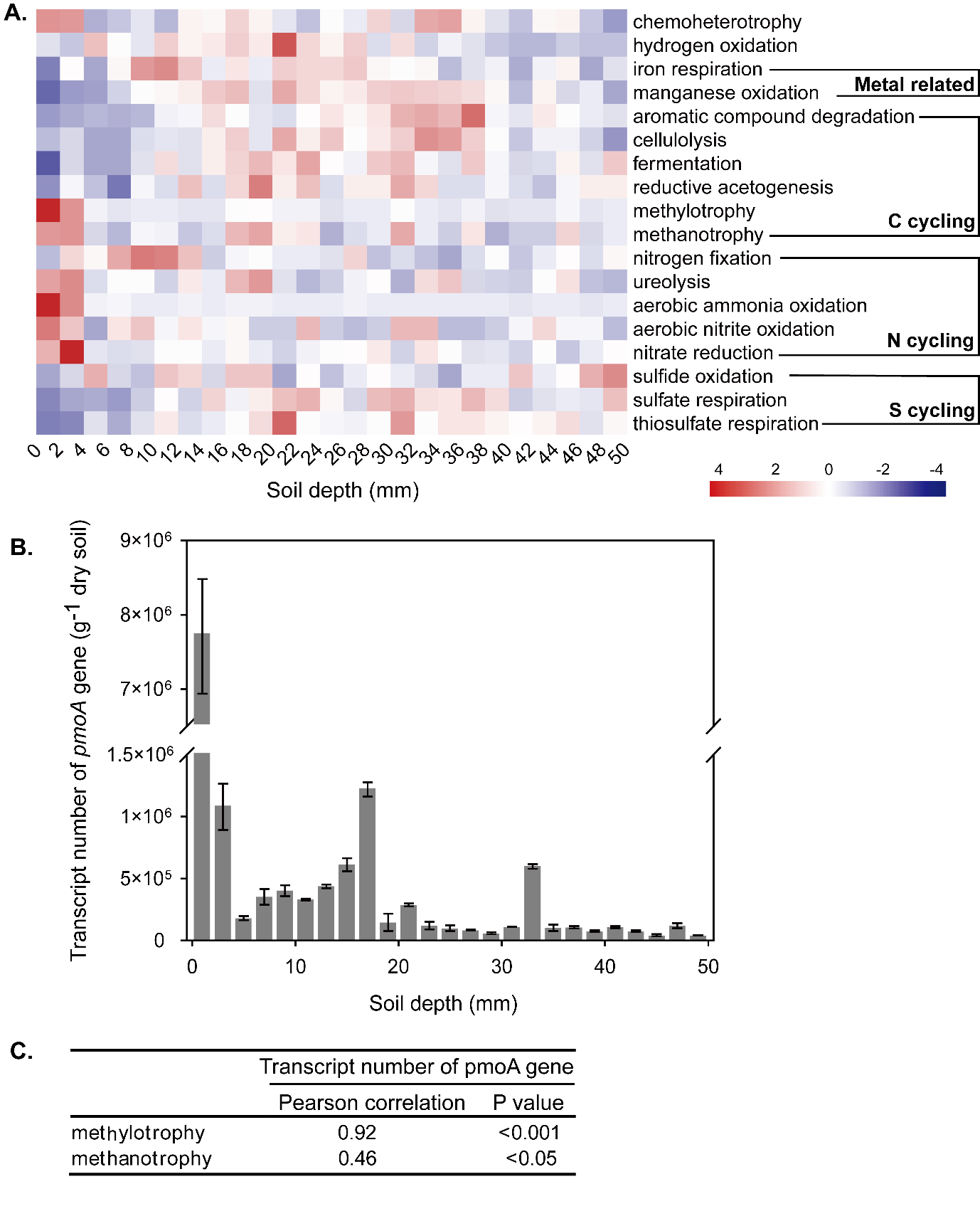


**Figure S3.** Putative metabolic functions of active bacteria across the soil-water interface. A) Functional community profile predicted from FAPROTAX. An increase in abundance tends toward red, while a decrease tends toward blue. B) Transcript copy number of *pmoA* (a functional marker for methane oxidizing bacteria. Error bar represents the measurement error. C) Pearson correlation analysis between *pmoA* gene transcript number and methane-oxidation relevant metabolic pathways.
